# A nonparametric Bayesian method of translating machine learning scores to probabilities in clinical decision support

**DOI:** 10.1186/s12859-017-1736-3

**Published:** 2017-08-07

**Authors:** Brian Connolly, K. Bretonnel Cohen, Daniel Santel, Ulya Bayram, John Pestian

**Affiliations:** 1 Department of Biomedical Informatics, Cincinnati Children’s Hospital Medical Center, 3333 Burnet Ave., MLC 7024, Cincinnati, OH 45229-3039 USA; 2 Computational Bioscience Program, University of Colorado School of Medicine, Denver, CO USA

**Keywords:** Statistics, Nonparametric, Bayesian, Calibration, Machine learning

## Abstract

**Background:**

Probabilistic assessments of clinical care are essential for quality care. Yet, machine learning, which supports this care process has been limited to categorical results. To maximize its usefulness, it is important to find novel approaches that calibrate the ML output with a likelihood scale. Current state-of-the-art calibration methods are generally accurate and applicable to many ML models, but improved granularity and accuracy of such methods would increase the information available for clinical decision making.

This novel non-parametric Bayesian approach is demonstrated on a variety of data sets, including simulated classifier outputs, biomedical data sets from the University of California, Irvine (UCI) Machine Learning Repository, and a clinical data set built to determine suicide risk from the language of emergency department patients.

**Results:**

The method is first demonstrated on support-vector machine (SVM) models, which generally produce well-behaved, well understood scores. The method produces calibrations that are comparable to the state-of-the-art Bayesian Binning in Quantiles (BBQ) method when the SVM models are able to effectively separate cases and controls. However, as the SVM models’ ability to discriminate classes decreases, our approach yields more granular and dynamic calibrated probabilities comparing to the BBQ method. Improvements in granularity and range are even more dramatic when the discrimination between the classes is artificially degraded by replacing the SVM model with an ad hoc k-means classifier.

**Conclusions:**

The method allows both clinicians and patients to have a more nuanced view of the output of an ML model, allowing better decision making. The method is demonstrated on simulated data, various biomedical data sets and a clinical data set, to which diverse ML methods are applied. Trivially extending the method to (non-ML) clinical scores is also discussed.

## Background

Clinical decision support systems can be defined as *any software designed to directly aid in clinical decision making in which characteristics of individual patients are matched to a computerized knowledge base for the purpose of generating patient-specific assessments or recommendations that are then presented to clinicians for consideration* [1, 2]. They are important in the practice of medicine because they can improve practitioner performance [1, 3–5], clinical management [6, 7], drug dosing and medication error rates [8–10], and preventive care [1, 11–16].

Machine learning (ML) gives computers the ability to learn from, and make predictions on the data without being explicitly programmed regarding the characteristics of that data [17]. It should not be surprising, then, that ML pervades clinical decision support, for two reasons. First, clinical decision support systems are structured such that patients are represented as features which can be used to map them to categories [18]. Second, healthcare data are complex - they can be distributed, structured, unstructured, incomplete, and not always generalizable.

Although logistic regression is widely used in biomedicine and it is highly recommended over ML approaches, ML algorithms have been used in many modern clinical decision support systems, ranging from predicting the incidence of psychological distress in Alzheimer’s Disease [19] to post-cardiac-arrest neuroprognostication [20]. A Google Scholar search of “machine learning biomedical” renders over 385,000 results.

However, there is a problem when ML algorithms are used for clinical decision support. The output of a ML model is usually a real number that is thresholded to produce a binary output. This outcome appears to come from a “black box”—a system module whose functioning is opaque. Yet, caregivers and patients prefer probabilistic statements [21–27]. But this “black box” approach runs counter to the goal of improving the decision-making power of physicians by providing more – not less – information to make better decisions [28]. In other words, “this patient has a 51% chance of developing heart disease” is more informative than a binary output of: “a ML algorithm has indicated that this patient belongs to a group of patients that develops heart disease.”

The effect of expressing clinical results probabilistically has been studied for decades. As early as 1977, Shapiro [29] introduced a method for assessing the predictive skills of physicians versus the results of “computerized procedures” that had been designed to provide probabilistic predictions of various clinical outcomes. Hopkins [30] suggested optimal plain-language descriptions of probabilities in a clinical setting. Grimes and Schulz [31] found that combining an accurate clinical diagnosis with likelihood ratios from ancillary tests improved diagnostic accuracy in a synergistic manner. Along these lines, Wells et al. [32] and Kanis et al. [33] provided specific examples of how probabilistic assessments of proximal deep vein thrombosis and bone fracture risk, respectively, could improve clinical outcomes.

Presenting results in probabilistic terms is as important to patients as it is to clinicians. Doctors using the decision-making probabilistic process will give information to patients about risks and benefits, often in numerical terms [34, 35]. Trevena et al. [36] found that patients have a more accurate understanding of risk if probabilistic information is presented as numbers rather than words, even though some may prefer receiving words.

The goal of this article is then to ensure that both patient and clinician can gain as much information as possible, and in the most straightforward way possible, from the output of an arbitrary ML algorithm by effectively converting ML-generated outputs to probabilities. The assumption here is that the clinician is uninterested in a simple cut-off, but wants to gain an intuitive sense to what degree the ML classifier “believes” that a datum belongs to one class or another. But for those who desire a threshold, the calibration is all the more important, since the rational choice of one class over the other is determined by whether the class probability is greater or less than 0.5.

There are three common calibration methods used to calibrate ML outputs to probabilities today: Platt Scaling [37], Isotonic Regression [38], and Quantile Binning, which are discussed in turn [39].

Platt’s method fits a logistic regression (LR) model to the ML scores from a training set, thereby providing an equation that directly transforms an ML-based classifier score to a probability. Although the LR model is not always appropriate and is prone to overfitting for small training sets, it can provide good calibration in certain circumstances (e.g., when Support Vector Machines are used as classifiers).

In an attempt to improve upon Platt’s method, the isotonic regression (IR) approach releases the linearity assumptions in the LR model, fitting a piece-wise constant non-decreasing function to the sorted ML scores in the training set. Although this calibration can yield good results, the isotonicity assumption is not always valid. In fact, Niculescu-Mizil and Caruana [40] demonstrated, using multiple classifiers and data samples of varying size, that both the Platt and IR methods can produce biased probability predictions.

Quantile Binning, on the other hand, mitigates the assumptions in the Platt and IR approaches by sorting the ML scores from a training set, and partitioning them into subsets (bins) of equal size. A new ML score can be simply transformed to a probability by locating its corresponding bin, and then calculating the fraction of positive outcomes in this bin from the training set [39]. While less restrictive than the other approaches, the drawbacks of this method include the fact that the number of bins must be set a priori, and that small training sets can corrupt the calibration. The Bayesian Binning in Quantiles (BBQ) method mitigates these limitations by effectively averaging over many binning schemes, which leads to a better overall calibration [41].

While it is difficult to argue with the overall accuracy and generalizability of the BBQ method, the present work will demonstrate that the granularity and dynamic range of calibrated probabilities, and in some cases the calibration accuracies, can be substantially improved by applying a novel non-parametric Bayesian approach. As with the previous methods, this approach requires a training set. But rather than using it to build a mapping between ML outputs and probabilities, the distributions of ML output from the positive and negative classes are directly compared to the ML output in question, rendering a probability that the ML output is derived from the one distribution versus the other.

Since the ML output is compared to the ML outputs of the two classes, a non-parametric approach is required, as there is no obvious binning strategy. Although there are many non-parametric Bayesian methods for comparing two-samples [42–45], non-parametric Bayesian methods for specifically quantifying the probability of distribution pairings (i.e., comparing the similarity of distribution A and B versus the similarity of A to C) are rare. Capitalizing on its power and simplicity, the Bayesian non-parametric two-sample comparison approach in Holmes et al. [46], is modified for this purpose. The improved calibration then arises from the non-parametric approach that effectively allows for an infinite number of binning schemes, and from naturally including statistical uncertainties due to finite training samples.

The methodology is tested on a variety of data sets that have been classified using two different ML techniques. It will be found that the method provides probability estimates with a high granularity within a broad range of calibrated probabilities. This is important for many clinical applications. For example, in risk assessment studies routinely performed by institutional review boards, government agencies, and medical organizations, it is crucial to be able to compute probabilities that are typically *<*1% [47–50]. Additionally, clinical literature abounds in examples where probabilities are expressed, or thresholds are determined, via plotting the logarithm of probabilities, to ensure interpretability at the extremes of the probability range [51–53].

## Methods

In the proposed approach, a binary ML classifier with a non-discrete score is assumed. It is further assumed that a training set is available, from which distributions of independent scores can be generated for the two classes in the data set. These distributions can be obtained by evaluating the score of the classifier applied to left out points during the leave-one-out (LOO) cross validation procedure. To determine the probability that a new datum is derived from a certain class, the ML classifier is evaluated for that datum. Then, a nonparametric Bayesian hypothesis test is applied to calculate the probability that the datum is derived from the parent distribution of that class as opposed to the parent distribution of the other class.

### Mathematical formalism

The (posterior) probability introduced above is calculated by modifying the formalism in Holmes et al. [46], which constructed a non-parametric Bayesian two-sample hypothesis test. In detail, suppose the probability that a single value *X*_p_ is derived from the parent distribution that generated a series of values X_1_, as opposed to the parent that generated values X_2_. The objective is to calculate *Pr*(*H*_1_ | *X*_p_ *,* X_1_ *,* X_2_), the posterior probability of the hypothesis *H*_1_ that *X*_p_ and X_1_ are derived from the same parent. The alternative hypothesis, *H*_2_, is that *X*_p_ is derived from the parent of X_2_. The probability of interest can then be expressed as

1 $$ \mathit{\Pr}\left({H}_1\Big|{X}_{\mathrm{p}},,,{\mathrm{X}}_1,,,{\mathrm{X}}_2\right)\propto \mathit{\Pr}\left({X}_{\mathrm{p}},,,{\mathrm{X}}_1,,,{\mathrm{X}}_2\Big|{H}_1\right)\mathit{\Pr}\left({H}_1\right). $$

where *Pr*(*X*_p_ *,* X_1_ *,* X_2_ | *H*_1_) is the likelihood of obtaining *X*_p_, X_1_, and X_2_ given that *X*_p_ and X_1_ are derived from the same parent distribution, and *Pr*(*H*_1_) is the prior probability for the hypothesis *H*_1_. The prior *Pr*(*H*_1_) is simply a number, containing a priori estimates of the occurrences of observations from class 1. The calculation of *Pr*(*X*_p_ *,* X_1_ *,* X_2_ | *H*_1_), on the other hand, is calculated with the help of Polya Trees [54].

Polya trees are a set Π of nested partitions in some space Θ. In this work, Θ is a one dimensional space where the ML scores are plotted. The partitions are generated by setting upper and lower bounds for the ML score derived from the training set, and then halving the space in several consecutive steps. At the start of the procedure, there is only “level 1” partitioning, where the two bins contain the number of score values, *N*_0_ and *N*_1_, that fall on each side of the partition. Each segment of the space is then halved again, producing a total of 4 bins for the “level 2” partitioning which contain the counts *N*_00_, *N*_01_, *N*_10_, and *N*_11_, and so on.

**Fig. 1:**
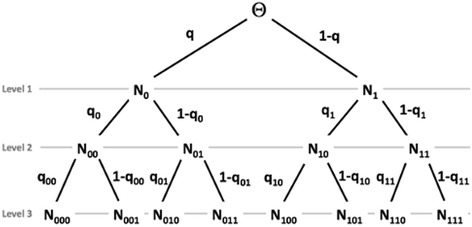
Construction of a Polya tree distribution. Adapted from Ferguson [54]

Figure 1 illustrates the partitioning and labeling of such counts in each bin. The *q*_X_'s indicate the probability of a value falling into the right vs. left partition. For instance, *q*_00_ is the probability of one of the *N*_00_ counts contained in bin ‘00′ falling into bin ‘000′ vs. bin ‘001′ at the next partitioning step.

*Pr*(*X*_p_ *,* X_1_ *,* X_2_ | *H*_1_) can then be constructed. Let us assume that the parent distribution for class 1 is described by some set of binomial parameters, *Q*. Likewise, suppose the parent distribution for class 2 is described by *R*, and *P* describes the parameters in the parent distribution of the “new” ML score. *P* is then equal to *Q* assuming hypothesis *H*_1_, and to *R*, assuming the alternative hypothesis *H*_2_. *X*_p_, X_1_ and X_2_ are realizations of *P*, *Q*, and *R*, respectively. Assume that, at the *j* ^th^ partition, *l*_j0_, *m*_j0_ and *n*_j0_ (*l*_j1_, *m*_j1_ and *n*_j1_) are the counts of values that fall on the left (right) side of the split in distributions *X*_p_, X_1_ and X_2_, respectively. The likelihood that *q*_j0_ (1 − *q*_j0_) at the *j* ^th^ partition is the same for distribution *P* and *Q*, but not *R*, is then:

2 $$ { \Pr}_j\left(\left.{\mathrm{X}}_p,,,{\boldsymbol{X}}_1,{\boldsymbol{X}}_2\right|{H}_1\right)=\int {dp}^{\hbox{'}} dpdqdr{ \Pr}_j\left({X}_p,,,{\boldsymbol{X}}_1,{\boldsymbol{X}}_2\left|{p}^{\hbox{'}}\right.,p,q,r{H}_1\right){ \Pr}_j\left({p}^{\hbox{'}},p,q,r{\left|H\right.}_1\right) $$

3 $$ =\int {dp}^{{\textstyle \hbox{'}}}dpdqdr\left[{p}^{\left({l}_{jo}\right)}{\left(1-p\right)}^{\left({l}_{j1}\right)}{q}^{\left({m}_{j0}\right)}{\left(1-q\right)}^{\left({m}_{j1}\right)}{r}^{\left({n}_{j0}\right)}{\left(1-r\right)}^{\left({n}_{j1}\right)}\right]\times \kern1em \left[\kern1em \begin{array}{c}\delta \left({p}^{{\textstyle \hbox{'}}}-p\right)\delta \left({p}^{{\textstyle \hbox{'}}}-q\right)\kern1em \\ {}\kern1em \frac{\varGamma \left({\alpha}_{j0}+{\alpha}_{j1}\right)}{\varGamma \left({\alpha}_{j0}\right)\varGamma \left({\alpha}_{j1}\right)}p{{\textstyle \hbox{'}}}^{\left({\alpha}_{j0}\right)-1}{\left(1-{p}^{{\textstyle \hbox{'}}}\right)}^{\left({\alpha}_{j1}\right)-1}\kern1em \\ {}\kern1em \frac{\varGamma \left({\alpha}_{j0}+{\alpha}_{j1}\right)}{\varGamma \left({\alpha}_{j0}\right)\varGamma \left({\alpha}_{j1}\right)}{r}^{\left({\alpha}_{j0}\right)-1}{\left(1-r\right)}^{\left({\alpha}_{j1}\right)-1}\end{array}\kern1em \right] $$

4 $$ \begin{array}{c}\hfill ={\left[\frac{\varGamma \left({\alpha}_{j0}+{\alpha}_{j1}\right)}{\varGamma \left({\alpha}_{j0}\right)\varGamma \left({\alpha}_{j1}\right)}\right]}^2\times \hfill \\ {}\hfill \frac{\varGamma \left({l}_{j0}+{m}_{j0}+{\alpha}_{j0}\right)\varGamma \left({l}_{j1}+{m}_{j1}+{\alpha}_{j1}\right)}{\varGamma \left({l}_{j0}+{l}_{j1}+{m}_{j0}+{m}_{j1}+{\alpha}_{j0}+{\alpha}_{j1}\right)}\times \hfill \\ {}\hfill \frac{\varGamma \left({n}_{j0}+{\alpha}_{j0}\right)\varGamma \left({n}_{j1}+{\alpha}_{j1}\right)}{\varGamma \left({n}_{j0}+{n}_{j1}+{\alpha}_{j0}+{\alpha}_{j1}\right)}.\hfill \end{array} $$

where Γ is the gamma function, *δ* is the Dirac delta function, { *α*_j0_ *, α*_j1_ } are parameters defined following a procedure described later in this section, and $\tilde{j}=\left\{\varnothing, 0,1,00,01,10,11,001,101,\dots \right\}$ (following the notation in Holmes et al. [46] and Fig. 1). Each *p*_∗0_, *q*_∗0_ and *r*_∗0_ are independently drawn from Beta(*α*_∗0_,*α*_∗1_).

Note the second set of brackets in Eq. 3 encompass the prior section which is comprised of two components: Dirac delta functions that act to tie *p* and *q* together through *p* ^′^, and terms involving gamma functions, which are Dirichlet priors.

Because each partition is assumed to be independent:

5 $$ \Pr \left({X}_{\mathrm{p}},{\mathrm{X}}_1,{\mathrm{X}}_2|{H}_1\right)=\begin{array}{c}\kern1em \\ {}\kern1em {\prod}_j\kern1em \end{array}{Pr}_j\left({X}_p,{\mathrm{X}}_1,{\mathrm{X}}_2|{H}_1\right) $$

*P* (*X*_p_ *,* X_1_ *,* X_2_ | *H*_2_) takes a similar form. With these two likelihoods, then, the posterior probability *P* (*H*_1_ | *X*_p_ *,* X_1_ *,* X_2_) can be calculated explicitly.

There are several practical considerations to keep in mind while calculating the posterior above. One is that the definition for *α*_X_ is adopted from Holmes et al. [46], where the *α*’s are set to be constant in a level such that *α*_L_ = *L* ^2^ = *α*_j0_ = *α*_j1_. Another point to consider is that floating point precision can lead to redundant score values. However, at least in the data sets considered in this work, stopping at the level where the values cannot be partitioned further is sufficient. In fact, it was found that in the data sets considered in this work, the number of levels could be limited to *<*19 without loss of calibration accuracy or granularity. However, it remains to be seen how generalizable this threshold might be.

The lower and upper bounds of the distribution also need to be determined. Holmes et al. [46] suggested partitioning in terms of quantiles. However, a more straightforward approach was found to be sufficient, where the partition is centered at the median of the training sample, and then expanding the upper and lower bounds of the partition space by equal amounts until it included all the points.

Lastly, priors on *H*_1_ and *H*_2_ are determined by the relative sizes of the classes in the training set.

### Comparing the BBQ method and the proposed approach

In this section, the method for generating reliability diagrams using a variety of data sets and ML classifiers to compare the state-of-the-art BBQ method and proposed method is described. Reliability diagrams [40, 55, 56] are generally used to evaluate the accuracy and granularity of the conversion methods by comparing the observed (true) frequency of an event with the predicted probability of an event. The predicted probabilities are discretely sorted into 10 bins, and for each bin, the mean predicted value is plotted against the true fraction of positive cases. The better the calibration, the closer the points will fall to the diagonal line. The finer the granularity, the more points (occupied bins) will be on the diagram.

The following two ML methods are used: a standard SVM-based classification method with a well-behaved, well understood score; and an ad hoc discriminant classification method constructed from a k-means algorithm.

The k-means discriminant is calculated by clustering a training set that contains two distinct classes of objects, and then determining which labels best represent each cluster. The centroid is determined for each cluster, and the label of a new (test) point is assigned via determining which centroid is proximal. Assuming two classes, *A* and *B*, the k-means discriminant is then defined as the ratio of the distances of the new point to the two centroids. (Along the same lines, the tuning of the SVM parameters and feature selection methods are also kept to a minimum to ensure a wide range of predicted probabilities for the reliability diagrams).

The unconventional definition of the k-means discriminant serves two purposes. First, the algorithm renders a classifier that has marginal performance, thereby allowing a better understanding of the proposed method’s behavior when there is a large overlap. Second, the k-means classifier output distributions are highly non-Gaussian, allowing insight into the proposed method’s generalizability.

The methods are demonstrated on three type of data sets: simulated classifier outputs, data sets from a popular ML data set repository, and a clinical data set. Each data set is divided into training and test subsets. The training sets are used to generate the distributions for the two classes, X_1_ and X_2_. The test sets are then used to create the reliability diagram, where each point in the test set, *X*_p_, is compared to X_1_ and X_2_ using both BBQ and the proposed method.

The simulated classifier outputs are generated from Gaussian distributions. The training set contains 50 positive cases randomly generated from a Gaussian distribution with zero mean and unit variance, and 50 negative cases are randomly generated from a second Gaussian distribution with a unit variance and certain fractional overlap with the first distribution (i.e., non-zero mean). With the BBQ and proposed method trained on these data, reliability diagrams are constructed on 100 test data with an equal number of positive and negative cases. The number of calibrated points in the reliability diagrams, range of predicted probabilities, and the goodness of fit of the calibrated points are evaluated. This training and testing is repeated 20 times for a given overlap in the Gaussian distributions and the results are averaged.

The biomedical data sets, described in Table 1, were taken from the University of California, Irvine Machine Learning repository [57, 58]. Although the balances between positive and negative instances vary dramatically between these data sets, any overfitting resulting from these imbalances would be accounted for in the calibration. To see this, suppose a ML algorithm produces an overfitted model if the data set is imbalanced. This imbalance is roughly approximated in the ‘training’ folds of the LOO cross-validation used to produce the distributions of positive and negative instances for the calibration. Any biases resulting from the ML algorithm’s tendencies to overfit are then accounted for in these distributions, since they are constructed from the test folds of the cross-validation.

**Table 1:** Description of the data sets obtained from the University of California, Irvine Machine Learning repository, including a brief description and the number of cases and controls in the training and testing sets used to demonstrate the proposed method

Data set	Description	Number of training Cases/Controls	Number of test Cases/Controls	Number of features	Citations
Lung Cancer	Clinical data, X-ray data, etc. used to predict 3 pathological types of lung cancer. The instances are divided into three classes of 9, 10, and 13 observations. For purposes here, the first two classes are aggregated into a single class.	8/8	11/5	54 integer clinical features	[66]
SPECT	Instances of normal and abnormal cardiac diagnoses.	40/40	172/15	22 binary features indicating partial diagnoses	[67, 68]
Parkinsons	Biomedical voice measurements from 31 people, including 23 with Parkinson’s disease.	72/25	75/23	22 real features	[69]
Arcene	Mass-spectrometric data that can be used to distinguish patients with cancer versus healthy subjects.	44/56	44/56	The data set contains 10,000 integer features; a Kolmogorov-Smirnov test [61] was used to choose the top 268 most discriminating features for classification.	[70]
Arrhythmia	Normal and “abnormal” instances of demographic and electrocardiogram features.	127/99	118/108	278 categorial, integer and real demographic and electrocardiogram features. A Kolmogorov-Smirnov test [61] was used to select the 32 most discriminating features for classification.	[71]
Breast Cancer	This data set contains features from a digitized images of fine needle aspirates (FNA) of breast masses, which describe characteristics of the cell nuclei present in the images. The data set contains benign and malignant instances of real-valued features.	130/219	111/239	8	[72, 73]
Contraception	This data set is a subset of the 1987 National Indonesia Contraceptive Prevalence Survey which samples married women who were either not pregnant or do not know if they were at the time of interview. The aim for the binary classifier constructed in this work is to predict whether or not a woman uses contraception based on their categorical and integer-valued demographic and socio-economic characteristics. The subset contains information for 1473 women, who are sub-divided based on their contraceptive use: no use (629), long-term methods (333), or short-term methods (511). The goal of the classifier is to classify women based on whether or not they use contraception based on categorical and integer-valued demographic and socio-economic characteristics.	423/313	421/316	8	[74]

The clinical data set, built to identify suicidal individuals using their language, contains the word frequencies of 161 suicidal and 153 control subjects from the Suicidal Adolescent Clinical Trial [59] and the Suicidal Thought Markers Study [60]. The data set contains 6226 unique words; a Kolmogorov-Smirnov test [61] was used to choose the top 124 most discriminating words for classification. The data with the reduced feature sets are L2 normalized on a per-subject basis to increase the discriminatory power of the SVM classifier and to therefore produce a wider range of ML scores.

The practical implementation of the proposed method is described in the previous section. The BBQ method implemented through the corresponding R package [62], using the default parameters and the “BDeu2” core function, as it was found to give finer granularity of probabilities for the SVM than “BDeu”. It was also found to give a far better calibration (although with fewer calibrated points) for the k-means algorithm on the Parkinson’s data set. However, the effect of changing these parameters will be explored.

## Results

**Fig. 2:**
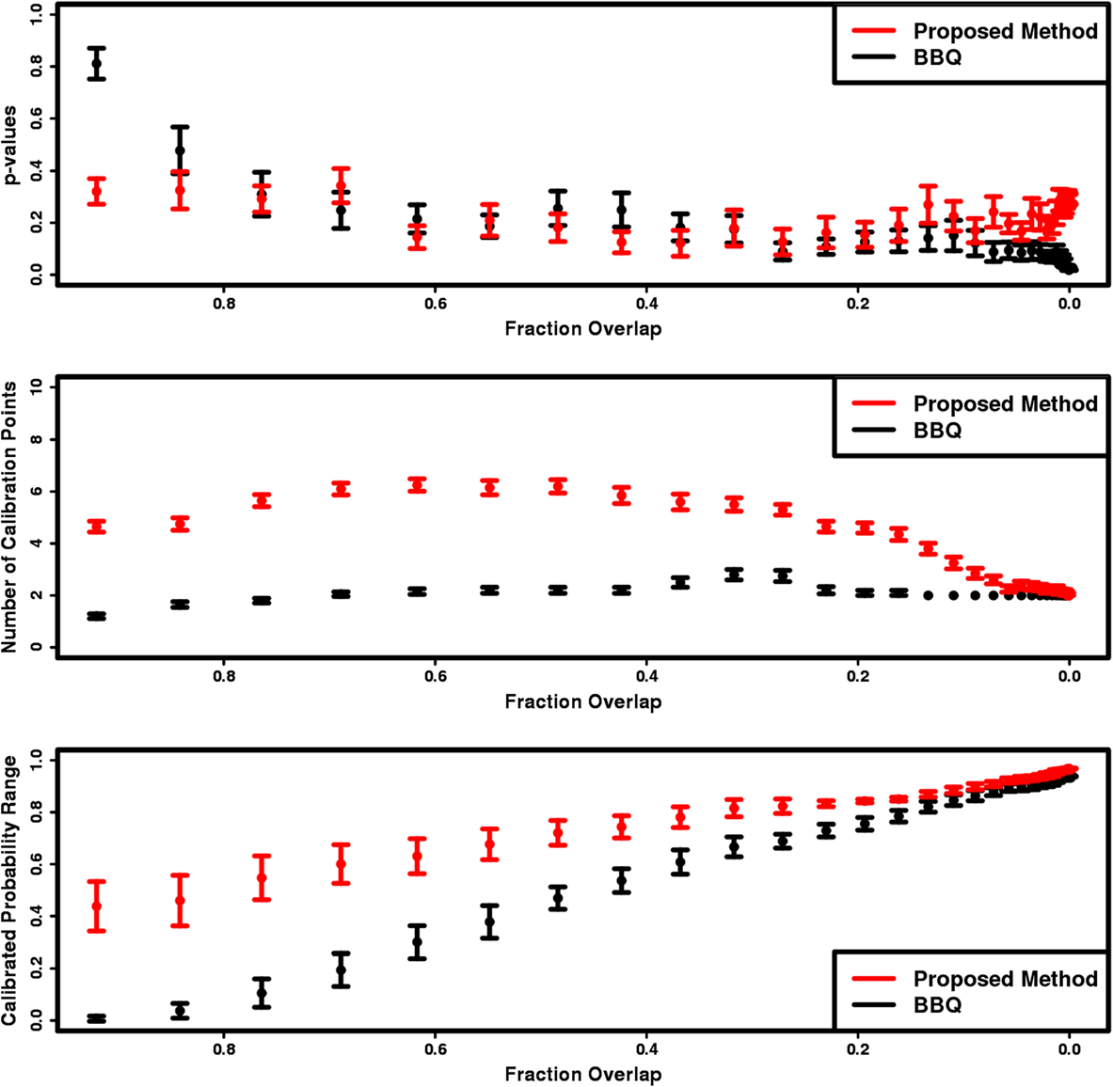
The averaged χ^2^ *p*-values from the fit of the calibration to the diagonal in the reliability diagrams (*top*), the average number of calibration points (*middle*), and the average range in calibrated probabilities (*bottom*) for the proposed method (*red*) and the BBQ method (*black*)

For the simulated data sets, reliability diagrams are constructed for various overlaps in the simulated ML output distributions. For a given overlap, the *χ* ^*2p*^-values quantifying the goodness of fit to a slope of 1, the number of calibration points, and the range in the calibrated probabilities are averaged and plotted. (The *χ* ^*2*^ is calculated by weighting the residuals by the inverse of the standard deviation of the calibrated probabilities). Figure 2 compares these averages as a function of the overlap. As evidenced by the *χ* ^*2*^ p-values, the calibration accuracies for the proposed method are comparable if not higher compared to the BBQ method, especially for smaller overlaps. The exception to this lies in the region of largest overlap, where the BBQ ethod outperforms the proposed method; however both methods produce fits with *p*-values greater than 0.2. Comparing the number of calibration points and calibrated probability ranges, it is clear the proposed method consistently outperforms the BBQ method.

**Fig. 3:**
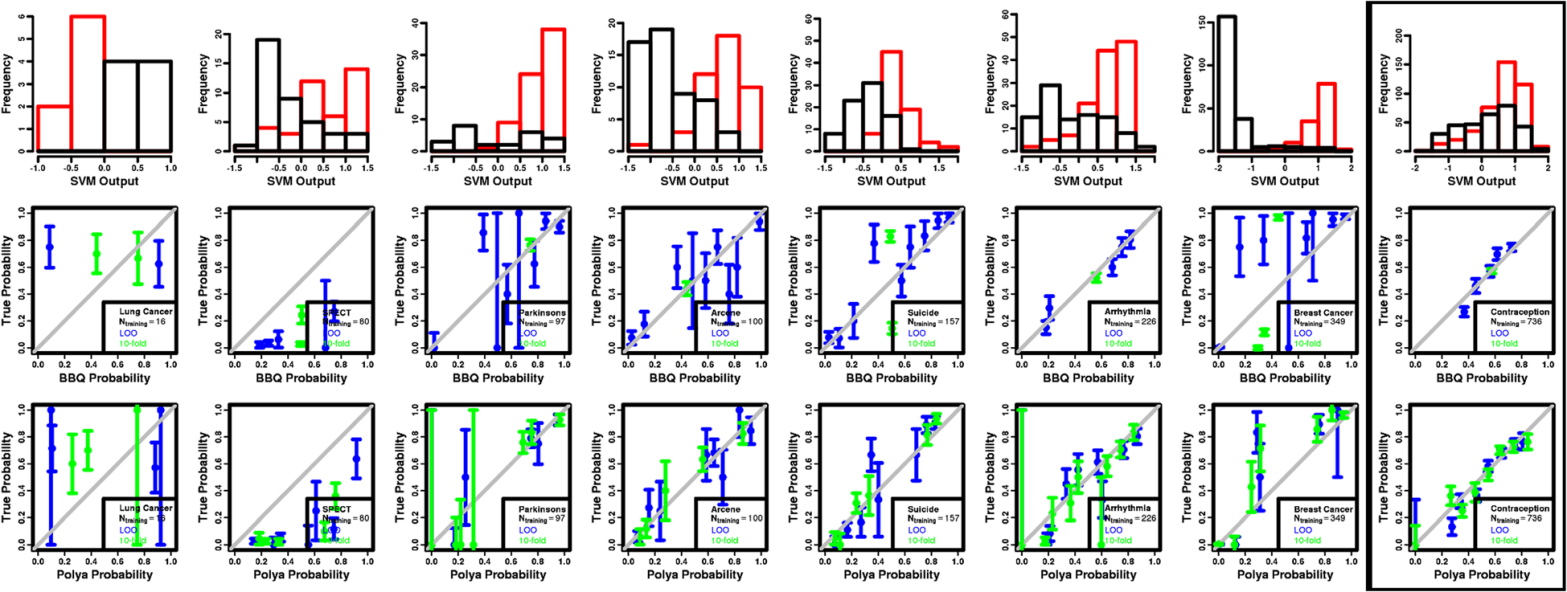
Histograms of SVM scores from the training set for the two classes, represented as *black* and *red* distributions (*top row*); reliability diagrams for the BBQ method (*middle row*), and for the proposed method (*bottom row*). For comparison, the training distributions are generated using both LOO (*blue*) and 10-fold cross validation (*green*). Those data sets with large overlaps between the predicted values from the two classes are *boxed for emphasis*. Note the larger granularity in the (*boxed*) data set with a larger overlap in the ML scores

**Fig. 4:**
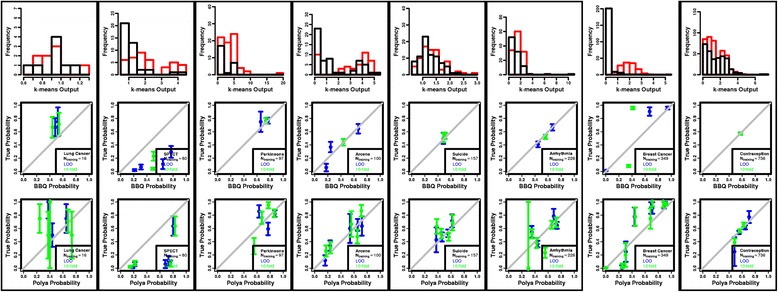
Histograms of k-means scores from the training set for the two classes, represented as *black* and *red* distributions (*top row*); reliability diagrams for the BBQ method (*middle row*), and for the proposed method (*bottom row*). For comparison, the training distributions are generated using both LOO (*blue*) and 10-fold cross validation (*green*). Those data sets with large overlaps between the predicted values from the two classes are* boxed for emphasis*. Note the systematically larger granularity in those (*boxed*) data sets with larger overlaps in the ML scores

But these results assume highly idealized (Gaussian) distributions for the ML outputs. Figures 3 and 4 then present the results from the biomedical data sets. They include the training set SVM and k-means ML scores used to generate the reliability diagrams, and the reliability diagrams themselves plotted with the diagonals indicating perfect calibration. For comparison, the training distributions are generated using both LOO and 10-fold cross validation. It can be seen changing the k-fold cross-validation used to build the training distributions simply leads to fewer calibration points for both BBQ and the proposed method.

Tables 2 and 3 show the *χ* ^2^ *p*-values and number of calibrated points for the SVM- and k-means- based classifiers, respectively, for both BBQ and the proposed method. One can see that the calibrations are, on average, comparable for the two methods. This is especially true when the ML scores from each class are unimodal and cleanly separated from the other class. Pair-wise t-tests between the *χ* ^2^ *p*-values yield *p*-values of 0.61 and 0.58 for the SVM and k-means classifiers, respectively. However, the advantages of the proposed method become apparent for larger overlaps in the class distributions of ML scores. This is shown by comparing the accuracies, numbers of calibrated points, and range of calibration points for the SVM and k-means method with more and less overlaps in the ML scores, respectively. Performing a pair-wise, one-sided t-test between the number of calibrated points for the two methods gives a *p*-value of 0.19 for the SVM classifier, where he overlaps are smaller, indicating the BBQ and the proposed method render similar numbers of calibrated points. However, performing a similar test with the k-means classifier where the overlaps are large gives a t-test *p*-value of 0.002, indicating the method renders a systematically larger number of calibrated points. Performing the same test on the ranges, the *p*-values are 0.06 and 0.01 for the SVM and k-means classifiers, respectively, indicating a systematically more dynamic range of calibrated probabilities. That is, the results are more dramatic when the tests are performed on just those data sets with high overlap, highlighted in Tables 2 and 3. While the t-test *p*-value for the *χ* ^2^ *p*-values indicates comparable calibration accuracies (0.67), the t-test *p*-values for the calibration points and ranges indicate substantial differences (0.0002 and 0.003, respectively). It can then be concluded that the proposed method renders a systematically larger number and more dynamic range of calibrated probabilities on the biomedical and clinical data sets. Note that, for either method, calibration does not seem to be affected by either sample size or the balance of the data set.

**Table 2:** The χ^2^ *p*-values for the fit to the diagonal in the reliability diagram, number of calibrated points, and difference between the maximum and minimum calibrated probabilities (range) for the SVM classifier presented in Fig. 3

Data set	BBQ			Proposed method		
	χ^2^ *p*-value	Calibrated points	Range	χ^2^ *p*-value	Calibrated Points	Range
Lung Cancer	<0.001	2	0.82	0.001	4	0.90
SPECT	<0.001	5	0.75	<0.001	7	0.92
Parkinsons	0.01	8	1.0	0.651	6	0.95
Arcene	0.387	9	0.96	0.841	8	0.94
Suicide	0.048	9	0.94	0.013	8	0.90
Arrhythmia	0.521	5	0.66	0.001	9	0.87
Breast Cancer	0.003	8	1.0	0.001	7	1.0
**Contraception**	**0.018**	**5**	**0.48**	**0.124**	**8**	**0.81**

The (Contraception) data set with a large overlap in the score distributions is emphasized in boldface. When compared with the other data sets, the proposed method produces a larger number of calibrated points, indicating a finer granularity in the calibrated probabilities

**Table 3:** The χ2 *p*-values for the fit to the diagonal in the reliability diagram, number of calibrated points, and difference between the maximum and minimum calibrated probabilities (range) for the k-means classifier presented in Fig. 4

Data set	BBQ			Proposed method		
	χ^2^ *p*-value	Calibrated points	Range	χ^2^ *p*-value	Calibrated points	Range
Lung Cancer	**0.087**	**2**	**0.27**	**0.038**	**3**	**0.62**
SPECT	<**0.001**	**4**	**0.75**	<**0.001**	**5**	**0.79**
Parkinsons	**0.544**	**2**	**0.11**	**0.006**	**3**	**0.28**
Arcene	**0.032**	**3**	**0.61**	**0.623**	**5**	**0.60**
Suicide	**0.497**	**2**	**0.05**	**0.724**	**4**	**0.34**
Arrhythmia	**0.389**	**2**	**0.26**	**0.012**	**4**	**0.43**
Breast Cancer	<0.001	3	0.96	<0.001	8	0.98
Contraception	**0.867**	**1**	**0.003**	**0.380**	**4**	**0.52**

The data sets with large overlaps in the score distributions are emphasized in boldface. The proposed method consistently achieves a larger number and more dynamic range of calibrated points. Note the Contraception data set has one calibration point on the reliability diagram, but a finite range. This is due to the number of calibration points being calculated from the number of (binned) points in the reliability diagram

Although Naeini et al. [41] suggested optimum parameters for the BBQ method. It is worth exploring whether the comparisons with the proposed method may change if they are altered. The scoring method, binning (*N*0), and the threshold that determines the optimal binning (*α*) are then modified and the BBQ method is re-evaluated on one of the data sets (the clinical data set) to gauge the parameters’ effect on the calibration. Table 4 shows the calibration points, range of calibration points, and reliability diagrams as a function of the changing BBQ parameters. It is clear from Table 4 that dramatically altering the BBQ parameters does not strongly effect the calibration for either the SVM or k-means classifiers.

**Table 4:** The χ^2^ *p*-values for the fit to the diagonal in the reliability diagram, number of calibrated points, and difference between the maximum and minimum calibrated probabilities (range) for various BBQ parameters Fig. 3

Classifier	Scoring function	Threshold (*α*)	Binning parameter (*N* 0)	_*χ*_2 *p*-value	Calibration points	Range
SVM	BDeu	2	0.0001	0.187	7	0.95
SVM	BDeu	4	0.0001	0.13	8	0.97
SVM	BDeu2	N/A	0.0001	0.023	9	0.97
SVM	BDeu	2	0.001	0.187	7	0.95
SVM	BDeu	4	0.001	0.13	8	0.97
**SVM**	**BDeu2**	**N/A**	**0.001**	**0.048**	**9**	**0.94**
SVM	BDeu	2	0.01	0.187	7	0.95
SVM	BDeu	4	0.01	0.13	8	0.97
SVM	BDeu2	N/A	0.01	0.066	9	0.94
k-means	BDeu	2	0.0001	0.502	2	0.05
k-means	BDeu	4	0.0001	0.558	2	0.06
k-means	BDeu2	N/A	0.0001	0.497	2	0.05
k-means	BDeu	2	0.001	0.502	2	0.05
k-means	BDeu	4	0.001	0.558	2	0.06
**k-means**	**BDeu2**	**N/A**	**0.001**	**0.497**	**2**	**0.05**
k-means	BDeu	2	0.01	0.502	2	0.05
k-means	BDeu	4	0.01	0.558	2	0.06
k-means	BDeu2	N/A	0.01	0.496	2	0.05

The BBQ default parameters used in the comparisons above are highlighted in boldface

## Discussion

In this work, a novel method for calibrating ML scores to probabilities was introduced. Using a number of data sets of varying sizes and two different ML methods, it was demonstrated that this method allows a more granular and more dynamic range of calibrated probabilities as compared to a current state-of-the-art calibration technique (BBQ). This is not surprising given that, unlike the BBQ, our method is not limited to a finite set of binning schemes for the calibration, and it naturally folds in statistical uncertainties due to the limited size of the training sample. Also, the proposed method systematically pushes out the upper and lower boundaries of the calibrated probabilities, allowing for more extreme (dynamic) probabilities, which are crucial for assessing clinical risk. The advantages of the proposed method are particularly dramatic in the 8 cases boxed in Figs. 3 and 4, where the overlaps between the class distributions of ML scores becomes large. The results from the simulated data indicate that high accuracies in calibration are possible, especially when the overlaps in the ML score of the two classes are small.

Further, as evidenced by the results from the Lung Cancer, Parkinsons, Suicide, Arrhythmia, Breast Cancer and Contraception data sets, the imbalance of the train or test data sets do not have an effect on the accuracy of the calibration. Sample size also does not appear to strongly affect calibration either.

It is also interesting that both the proposed method and the BBQ method were trained using ML output distributions generated from LOO cross-validation of the training set that was used to generate the ML model. The same training set was therefore used to train both the calibration method and the ML model, and both calibration techniques were able to calibrate the ML scores to a high overall accuracy. That is, the results suggest separate data sets might not be necessary to train the model and build the case and control distributions for the calibration. Decreasing the number of folds only decreases the granularity of both the BBQ and the proposed method, as demonstrated in Figs. 3 and 4.

In summary, the results indicate that the proposed method gives comparable or better accuracy (as indicated from the simulated ML outputs). Both the simulated and real data sets indicated a systematic finer granularity and greater range of calibrated probabilities using the proposed method, especially when there are large overlaps in the ML output distributions for the two classes. Tests on the clinical data set indicate changes in the BBQ parameters would not change these conclusions.

However, questions may remain as to why ML methods that return a non-probabilistic result should be considered when there are so many probabilistic ML methods in the literature. For instance, in Sowa et al. [63], logistic regression (LR), decision tree (DT), support-vector machine (SVM), and random forest (RF) models were trained to distinguish between individuals with non-alcoholic non-fatty liver disease (NAFLD) and alcoholic non-fatty liver disease without cirrhosis (ALDNC), and between alcoholic liver disease with cirrhosis (ALDC) and alcoholic liver disease without cirrhosis ALDNC. All of the ML models yielded comparable accuracies, with the RF carrying the advantage of a probabilistic interpretation. There would still be advantages to converting the ML scores to probabilities in this case. For instance, as shown in Malley et al. [64], the probabilities returned by these models – including the LR and RF ones – cannot necessarily be taken at face value. Also, our method acts to normalize the ML results from the four classifiers onto a single, intuitive scale. But, more broadly, there are instances where ML models with non-probabilistic outputs outperform methods that allow a probabilistic interpretation of the results. For instance, Statnikov et al. [65] compared RF and SVM models for microarray-based cancer classification, finding that SVM models consistently outperformed RF models.

## Conclusions

A novel non-parametric Bayesian technique is proposed for calibrating the outputs of an ML-based algorithm to a probability. The method’s generalizability was demonstrated by applying it to two disparate ML classifier discriminants: an SVM discriminant and an arbitrarily defined k-means discriminant. In applying this method to these classifiers over a diverse array of real and simulated data sets, it was shown to yield a broader, more dynamic range of calibrated probabilities with a finer granularity, especially when discrimination between the classes is poor. This provides more nuanced diagnostic and prognostic probabilistic assessments from ML-based clinical decision support systems, allowing clinicians and patients to make better decisions. Therefore, converting ML outputs to probabilities substantially improves clinical decision making.

Although the focus of this work has been calibrating ML scores, there is no reason why the output necessarily needs to be derived from a machine. It can easily be extended to calibrate any clinical score (e.g., a psychiatric rating scales, illness severity scores, etc.), where the prior on *α*_L_ goes as 2^−L^ if the scores are discrete [46].

In future work, methods of generalizing this formalism to multi-class problems will be explored. This is not a trivial undertaking, as many scores may need to be combined to calculate a posterior probability. Other future research directions will include understanding how the Bayesian formalism might be leveraged to include hypotheses which assume that the new (test) point *X*_p_ is not derived from either of the parent class distributions.

## Abbreviations

BBQ: Bayesian binning in quantiles
IR: Isotonic regression
LOO: Leave one out
LR: Logistic regression
ML: Machine learning
STM: Suicide thought markers
